# The impact of social networks on the behavior of households’ participation in rural environmental human settlement improvement: evidence from Jiangxi Province, China

**DOI:** 10.3389/fpsyg.2025.1697253

**Published:** 2025-11-17

**Authors:** Chao Chen, Ruohan Peng, Feng Ye, Yang Liu

**Affiliations:** 1 School of Economics and Management, Jiangxi Agricultural University, Nanchang, China; 2 Business School, The University of New South Wales, Sydney, NSW, Australia; 3 Rural Revitalization Strategy Research Institute, Jiangxi Agricultural University, Nanchang, China; 4 School of Economics, Inner Mongolia University of Finance and Economics, Hohhot, China

**Keywords:** environmental remediation, neighborhood relationships, cadre-mass relationships, ecological cognition, place attachment

## Abstract

Promoting rural human settlement improvement and improving the quality of rural livability is an important part of enhancing farmers’ well-being. Based on the survey data of 512 farm households in Jiangxi Province, this study uses the multivariate ordered probit model and the mediation effect model to reveal the influence of social networks on the behavior of households’ participation in rural human settlement improvement and the mechanism of influence. The results show that the social networks can significantly mobilize households’ enthusiasm to participate in rural human settlement improvement, and the promotion effect of cadre-mass relationships in the social network is more evident than in neighborhood relationships. Social networks are more effective in promoting rural residents’ participation in environmental remediation among males, better-off families and non-plain areas. Ecological cognition and place attachment partially mediate the social networks to promote households’ participation in rural habitat improvement. Consequently, the government should encourage rural households to participate in environmental improvement by strengthening the construction of their social networks, improving their ecological knowledge, cultivating their place attachments and increasing the publicity of environmental improvement.

## 1 Introduction

The United Nations Sustainable Development Goals (SDGs) state that ensuring healthy lifestyles that promote the well-being of all people at all ages is essential for sustainable development. The quality of rural habitats not only directly affects the health of rural residents (Usman et al., 2019), but is also a key component in achieving the Sustainable Development Goals (SDGs). Nonetheless, sanitary infrastructure in many rural regions of China remains insufficient (Zhang et al., 2020). At the same time, problems such as garbage disposal, sewage flow, and poor toilet sanitation are prominent, which not only affect the appearance of villages, but also breed a large number of germs, threatening the health of rural residents (Wang et al., 2017; Guo et al., 2021). Therefore, improving the rural living environment and building harmonious and livable villages are key elements in the implementation of China’s rural revitalization strategy. Since the implementation of a number of rural habitat improvement programs, various regions have taken active action to effectively promote the comprehensive development of rural habitat improvement work, and the persistent unsanitary and disordered circumstances in rural regions have been markedly enhanced. As of 2022, the sewage treatment rate in rural China has attained around 31% (Cao et al., 2024), the penetration rate of sanitary latrines exceeded 73%, and more than 90% of natural villages had established effective domestic waste collection and disposal systems. These improvements have enhanced rural habitats and substantially increased the sense of well-being and happiness among rural residents.

Despite these achievements, the overall quality of rural habitat improvement still requires significant enhancement (Fang et al., 2023). Current efforts have yet to meet the standards necessary for the modernization of agriculture and rural areas, nor do they fully satisfy rural residents’ aspirations for an improved quality of life. China’s ecological civilization construction has reached a critical stage, but substantial challenges remain in environmental protection. The lack of technical and economic policy support continues to constrain the effectiveness of environmental remediation (Jaffe et al., 2005), while weak environmental awareness and low participation rates among rural residents hinder further progress (McGurk et al., 2020). As key stakeholders, rural residents play a central role in improving their habitats. However, rural environmental management, as a form of public resource governance, relies heavily on collective action. Mobilizing farmers’ enthusiasm, initiative, and creativity in environmental governance is critical to sustaining long-term improvements (Canessa et al., 2024).

The development of rural civilization activities has fostered increased interaction among farmers and between farmers and village officials (Lee et al., 2021). These strengthened social networks have not only enhanced social cohesion but also raised questions about their potential to motivate farmers to engage more actively in environmental remediation. Thus, understanding the impact of social networks on farmers’ engagement in rural habitat improvement and the underlying mechanisms has become a pressing issue in building “Beautiful Villages” in China.

Extensive academic research has explored the factors influencing rural habitat improvement. Prior studies have examined how farmers’ participation in activities such as waste management, sewage treatment, and sanitary latrine reforms is driven by different causes, which may be roughly classified into external and internal drivers. External factors include environmental regulations, policies, and policy tools. For example, different forms of environmental regulation—such as incentive-based, coercive, and guiding approaches—have been shown to enhance farmers’ readiness to participate in waste management (Xu et al., 2023). The enactment of environmental policies will regulate the pro-environmental behavior of residents (Kvakkestad et al., 2021). Policy tools (infrastructure improvement) will incentivize households to convert their waste separation intentions into actions (Govindan et al., 2022).

Internal factors, on the other hand, encompass individual characteristics, psychological cognition, and situational influences. Demographic attributes, such as gender, age, education, health status, and employment, significantly affect farmers’ engagement in habitat improvement (Vu et al., 2020; Unay-Gailhard and Bojnec, 2021; Savari et al., 2023). Psychological constructs, including environmental awareness (Qing et al., 2022), and responsibility (Jia et al., 2021) also shape behaviors such as household waste sorting and toilet reforms. Place attachment (Valizadeh et al., 2020) and self-identity (Cullen et al., 2020) also exerts considerable beneficial influence on pro-environmental behaviors of farmers. Among the situational factors, social capital—including social networks, prestige, and participation—and consumption habits have significant effects on farmers’ domestic waste disposal practices (Deng et al., 2022; Cao et al., 2023).

Social capital has been widely recognized for its positive impact on sustainable agricultural and rural development (Rivera et al., 2019). In the context of rural habitat improvement, social capital—including structural, relational, and innovative components—significantly enhances environmental performance (Zhou et al., 2020). For instance, social networks comprising social norms, trust, and interpersonal connections positively influence farmers’ willingness and behaviors to participate in waste management and actively participate in waste classification (Wang et al., 2022; Zhang et al., 2023; Teng et al., 2024). Improved social networks further promote environmental remediation behaviors, with environmental awareness serving as a mediating factor (Xie et al., 2021). Additionally, clan networks facilitate participation, with the authority of village leaders playing a moderating role (Xu and Xia, 2023). Similarly, strong farmer-official relationships increase farmers’ willingness to manage domestic waste and maintain village water bodies (Jian et al., 2024).

While previous studies have established a foundation for understanding the factors influencing rural habitat improvement, several gaps remain. First, rural habitat improvement involves a diverse range of behaviors, yet most studies have focused narrowly on specific activities, neglecting broader patterns of participation (Jian et al., 2024; Teng et al., 2024). Second, the roles of ecological cognition and place attachment—two critical mediators in the relationship between social networks and environmental behavior—have received limited attention (Zhang et al., 2023; Jian et al., 2024). Third, existing studies often employ one-dimensional or vague measures of social networks, ecological cognition, and place attachment (Wang et al., 2021; Wang and Zhang, 2023; Xu and Xia, 2023), focusing primarily on single indicators such as neighborly relations or cadre-farmer interactions. Finally, the differential impacts of neighborhood and cadre-mass relations on farmers’ involvement in environmental remediation remain underexplored.

To address these gaps, this study investigates the impact of social networks on farmers’ involvement in rural habitat enhancement using survey data collected from Jiangxi Province in 2021. Employing a multivariate ordered probit model and mediation analysis, the study examines the impact of social networks on participation behaviors, specifically emphasizing the mediating impacts of ecological cognition and place attachment. Additionally, it explores the distinct impacts of neighborhood relations and cadre-mass relations. The findings aim to provide targeted recommendations for enhancing rural households’ engagement in improving their living environments.

This study contributes to the literature in four significant aspects. First, it expands the scope of existing research by examining the impact of social networks on farmers’ diverse environmental behaviors. Second, it sheds light on the mediating roles of ecological cognition and place attachment, offering insights into the mechanisms underlying these relationships. Third, it adopts a multidimensional approach to measure social networks, ecological cognition, and place attachment. Finally, it highlights the differential impacts of neighborhood and cadre-mass relations, providing nuanced insights into their roles in rural environmental governance.

The subsequent sections of this paper are organized as follows: Section 2 comprises the theoretical examination and formulation of research hypotheses. In Section 3, the materials and procedures are presented. The findings and discussion are presented in Section 4, while the conclusions and recommendations are provided in Section 5.

## 2 Theoretical analysis and research hypotheses

### 2.1 Impact of social networks on farmers’ participation in rural human settlement improvement

According to the theory of social embedding, farmers’ decisions are inherently influenced by the social networks in which they are situated. In rural China, communities are shaped by strong geographical and kinship ties, forming an acquaintance-based society. These social networks profoundly affect the production and life decisions of farmers (Michelini, 2013; Chaudhuri et al., 2021). Consequently, farmers’ environmental remediation behaviors are significantly shaped by their social networks (de Krom, 2017; Zhou et al., 2022).

In rural settings, the primary components of farmers’ social networks include neighborhood relationships and cadre-mass relationships. Neighborhood relationships refer to the interactions and trust between farmers living in geographical proximity. Such relationships are pivotal in fostering social cohesion and harmony within rural communities. Cadre-mass relationships, on the other hand, encompass the connections and trust between farmers and village cadres. As a unique form of social capital, cadre-mass relationships often serve as an endogenous resource for village cadres to mobilize during governance efforts (Jian et al., 2024). A robust social network helps motivate farmers to participate actively in environmental remediation (Xie et al., 2021).

First, within social networks, individual attitudes and behaviors are disseminated, exerting a demonstrative and mobilizing effect on surrounding individuals. This dynamic is particularly evident among village cadres, who, as rural elites, assume a prominent leadership role in creating a favorable environment for remediation efforts. Their influence fosters a collective remediation ethos, motivating farmers to participate actively in environmental initiatives (Peng et al., 2024).

Second, harmonious social networks facilitate the development of informal, mutually beneficial systems among farmers. These systems strengthen group identity and responsibility, promoting collective engagement in environmental remediation. Moreover, strong cadre-mass relationships enhance collaboration and mutual commitment between the government and farmers. This alignment increases the likelihood of institutional rule adoption and adherence, thereby promoting the maintenance and enforcement of environmental remediation practices (Hao et al., 2022).

Consequently, this study proposes Hypothesis 1: social networks will significantly promote farmers’ participation in rural habitat improvement.

### 2.2 Analysis of the mechanism of the influence of social networks on the behavior of rural human settlement improvement

#### 2.2.1 Social networks, ecological cognition, and rural human settlement improvement behavior

Behavioral economics posits that individual cognitive level significantly determines decision-making behavior. Specifically, an increased level of ecological cognition correlates with a greater desire among farmers to engage in environmental rehabilitation efforts. Ecological cognition pertains to an individual’s awareness and comprehension of the state of the surrounding ecosystem and its changes and also includes knowledge of environmental policies. In the lives of farm households, this cognition is crystallized in how they understand and assess the current rural ecosystem and how they understand and respond to environmental policies. This cognition not only shapes farmers’ choices about agricultural production and lifestyles but also reflects whether they recognize the significance of environmental conservation and have a sense of responsibility to protect the environment. A greater level of ecological cognition among farmers correlates with an increased propensity to engage in ecologically sustainable practices that promote agricultural growth and ecological conservation (Qing et al., 2022).

This study measures ecological cognition in terms of conceptual, value, and responsibility cognition. A higher level of farmers’ knowledge of policy concepts and environmental knowledge promotes pro-environmental behavior (Kurokawa et al., 2023). Farm households possessing elevated value perception are more inclined to demonstrate pro-environmental behaviors (Ngoc et al., 2024). Awareness of environmental responsibility significantly promotes farmers’ participation in domestic trash segregation (Jia et al., 2021).

To improve farmers’ ecological cognition, a stable and reliable information channel is needed. For farmers, the social networks is a more stable information acquisition channel. Social cognition theory emphasizes the enormous influence of the social environment on individual cognition, and social networks, as a unique manifestation of the social environment, markedly affecting the development of farmers’ ecological cognition (Wan and Du, 2022). Generally speaking, neighborhood relationships and cadre-mass relationships can expand the avenues via which farmers get information. Communication among farmers can improve their knowledge of environmental policies and their appreciation for the value of environmental protection. In addition, the policy propaganda of the village committee is also one of the important ways for farmers to understand the policy, and the close cadre-mass communication can enhance the farmers’ knowledge of the policies, regulations and village rules and regulations related to environmental remediation. At the same time, a high degree of trust in the village cadres will enhance their recognition of the policies, regulations and village rules, stimulate their sense of responsibility for environmental protection, and make them actively participate in environmental remediation actions that are beneficial to both themselves and the village community.

Consequently, this study proposes Hypothesis 2: Ecological cognition mediates the role of social networks in motivating farmers to participate in rural habitat improvement.

#### 2.2.2 Social networks, place attachment and rural human settlement improvement behavior

Place attachment refers to the emotional identification of farmers with the village and villagers because they have lived there for a long time, which shows their importance and love for their hometown (Pei, 2019) place attachment can significantly promote farmers’ participation in environmental remediation (Buta et al., 2014; Song and Soopramanien, 2019). From a psychological perspective, when farmers develop a profound sense of belonging to the village, this sense of belonging can gradually weaken the individual’s “selfish” psychological tendency, which will make farmers prioritize the collective interests and actively participate in the improvement of human settlements (Ma et al., 2022; Soopramanien et al., 2023). From a human geography perspective, place attachment is a significant manifestation of the emotional bond between farmers and the geographic environment. This connection helps farmers recognize their strong connection with the environment, become more concerned about its state, and adopt pro-environmental behaviors.

Farmers’ social networks are an important influence on their place attachments. Social networks such as neighborhood relationships and cadre-mass relationship constitute the social interaction framework of farmers, in which farmers form a specific emotion toward the village through exchanging information, sharing resources, and interacting emotions (Kim et al., 2016; Luo et al., 2022). Specifically, by interacting with neighbors and village cadres, farmers can get a profound comprehension of the dynamics of village development and policy changes and build deep interpersonal trust, thus gradually forming an emotional identity with the village and villagers.

Consequently, this study proposes Hypothesis 3: place attachments mediates the role of social networks in motivating farmers to participate in rural habitat improvement.

**Figure 1:**
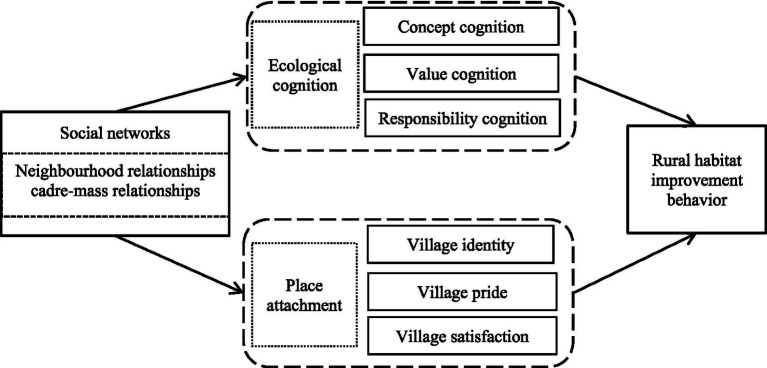
Theoretical analysis framework.

According to the aforementioned theoretical analysis, this study constructs a research model of social networks (including neighborhood relationships and cadre-mass relationships), ecological cognition (including concept cognition, value cognition, and responsibility cognition), and place attachment (including village identity, village pride, village satisfaction) on rural human settlement environment remediation behavior. The theoretical analysis framework is shown in Figure 1.

## 3 Materials and methods

### 3.1 Models and variables

#### 3.1.1 Ordered probit model

The explained variable of this study is the participation of rural households in rural habitat improvement behavior. Referring to related research (Xu and Xia, 2023), the following five measures with high adoption rate, high representativeness, and highly relevant to rural life are selected to characterize the explanatory variables, specifically including the use of flush toilets, harmless treatment of toilet feces, resource utilization of toilet feces or animal and poultry manure, domestic garbage disposal, and domestic sewage treatment behavior. Finally, the number of farmers participating is used to characterize their habitat improvement behaviors. Because the explained variable belongs to the ordinal variable. In view of this, this study constructs an ordered probit model for estimation. The regression equation is:


$$Y=\lambda_{1}+\mathrm{cX}+g_{1}C+\varepsilon_{1}$$ (1)

In Equation 1, *Y* is the behavior of farmers participating in enhancing the rural living environment; *X* represents the social networks, and the social networks is sometimes called a social network, which refers to the relationship between social participants and their relationships. Rural residents are the leading group in China’s rural areas. Their social networks is a factor that cannot be ignored in understanding the rural social structure, rural residents’ decision-making behavior and rural quality of life. It is mainly reflected in family relations, relatives and friends, neighborhood and cadre-mass relations.

In light of theoretical analysis and data accessibility, this study draws on related studies (Hao et al., 2022; Cao et al., 2023) to use external relationships such as neighborhood and cadre-mass relationships to measure the relational network and employs the entropy method of assigning weights to calculate the composite index of relational networks. The entropy technique can provide the weights more accurately representative of the significance of the respective indicators inside the established indicator system; C is the control variable, selecting personal characteristics such as gender, age, educational level, health status, household characteristics such as family size, household economic status, village characteristics such as village road condition, village hygienic condition, topography, and regional variables for control (Jia et al., 2021; Bai and Lin, 2022); λ_1_ is a constant term; c, g_1_ denote the regression coefficients of each variable; and ε_1_ is a random disturbance term obeying a normal distribution.

#### 3.1.2 Modeling of impact mechanisms

To verify whether ecological cognition and place attachment mediate between the social networks and rural habitat improvement participation behavior. Drawing on related research (Baron and Kenny, 1986), this study employs the three-step test regression coefficient method to test the mediating effect and constructed the model as follows:


$$Y=\lambda_{1}+\mathrm{cX}+g_{1}C+\varepsilon_{1}$$ (2)

$$M=\lambda_{2}+\mathrm{aX}+g_{2}C+\varepsilon_{2}$$ (3)

$$Y=\lambda_{3}+c\prime X+\mathrm{bM}+g_{3}C+\varepsilon_{3}$$ (4)

In Equations 2–4, *M* is the mediating variable, including ecological cognition and place attachment. Ecological cognition is the cognitive level of ecological and environmental conservation; in order to measure the ecological cognition level of farmers more scientifically and comprehensively. Utilizing existing studies (Walder and Kantelhardt, 2018; Qing et al., 2022; Chen et al., 2024), this study measures the extent of farmers’ ecological cognition using the three-dimensional indicators of concept cognition, value cognition, and responsibility cognition and derives the “comprehensive ecological cognition level” of farmers using the entropy value method.

As a psychological perception, place attachment is manifested as farmers’ attention to and love for their villages. Based on data availability and related studies (Lee and Oh, 2018; Ma et al., 2022), this study characterizes farmers’ place attachments from three dimensions, namely village identity, village pride, and village satisfaction, and assigns them weights by using entropy method; λ_i_ is the intercept; ε_i_ is the random perturbation term; g_2,_ g_3,_ a, b, c´ are coefficients to be estimated.

#### 3.1.3 Robustness and endogeneity tests

To guarantee the robustness of the baseline regression outcomes, this study employs multiple approaches, including altering the methodological model, adjusting the sample size, and introducing supplementary control variables. First, recognizing that the dependent variable—environmental remediation behavior—is a discrete multivariate ordinal variable, the study applies the ordinary least squares (OLS) method as an alternative to validate the robustness of the findings. Second, acknowledging that the majority of respondents are middle-aged or older individuals, whose cognitive and behavioral abilities may vary with age, the study re-estimates the baseline regression using a restricted sample of respondents aged 30–70 years. Third, given the potential influence of garbage collection facilities on farmers’ participation in environmental remediation, the study incorporates the availability of such facilities as an additional control variable. This adjustment accounts for the possibility that farmers in villages with better garbage collection infrastructure may demonstrate a higher propensity to engage in environmental remediation activities (Meng et al., 2022).

Addressing endogeneity concerns, the study recognizes the potential for bidirectional causality. On one hand, improved social networks among farmers are likely to enhance their enthusiasm for engaging in environmental remediation. On the other hand, participation in remediation activities may foster more harmonious neighborhood and cadre-mass relationships, thereby enhancing the overall quality of social networks.

To address this, the study draws on established methodologies (Tregear and Cooper, 2016; Hartmann et al., 2023) and utilizes the non-governmental organizations (NGOs) such as cooperative organizations, community service organizations, and recreational organizations in the village as an instrumental variable. In terms of relevance, NGOs provide a platform for communication and interaction among farmers, and village cadres may also be involved in order to strengthen the orderly development of the organization, which is conducive to broadening the social network of farmers, so there is a certain degree of relevance between the two. In terms of exogeneity, NGOs do not directly influence the environmental remediation behaviors of farmers themselves, which meets the requirement of exogeneity of instrumental variables. Based on these considerations, the study concludes that the selected instrumental variable is both relevant and appropriate for addressing endogeneity concerns.

### 3.2 Data collection and descriptive statistics

#### 3.2.1 Data collection

**Figure 2:**
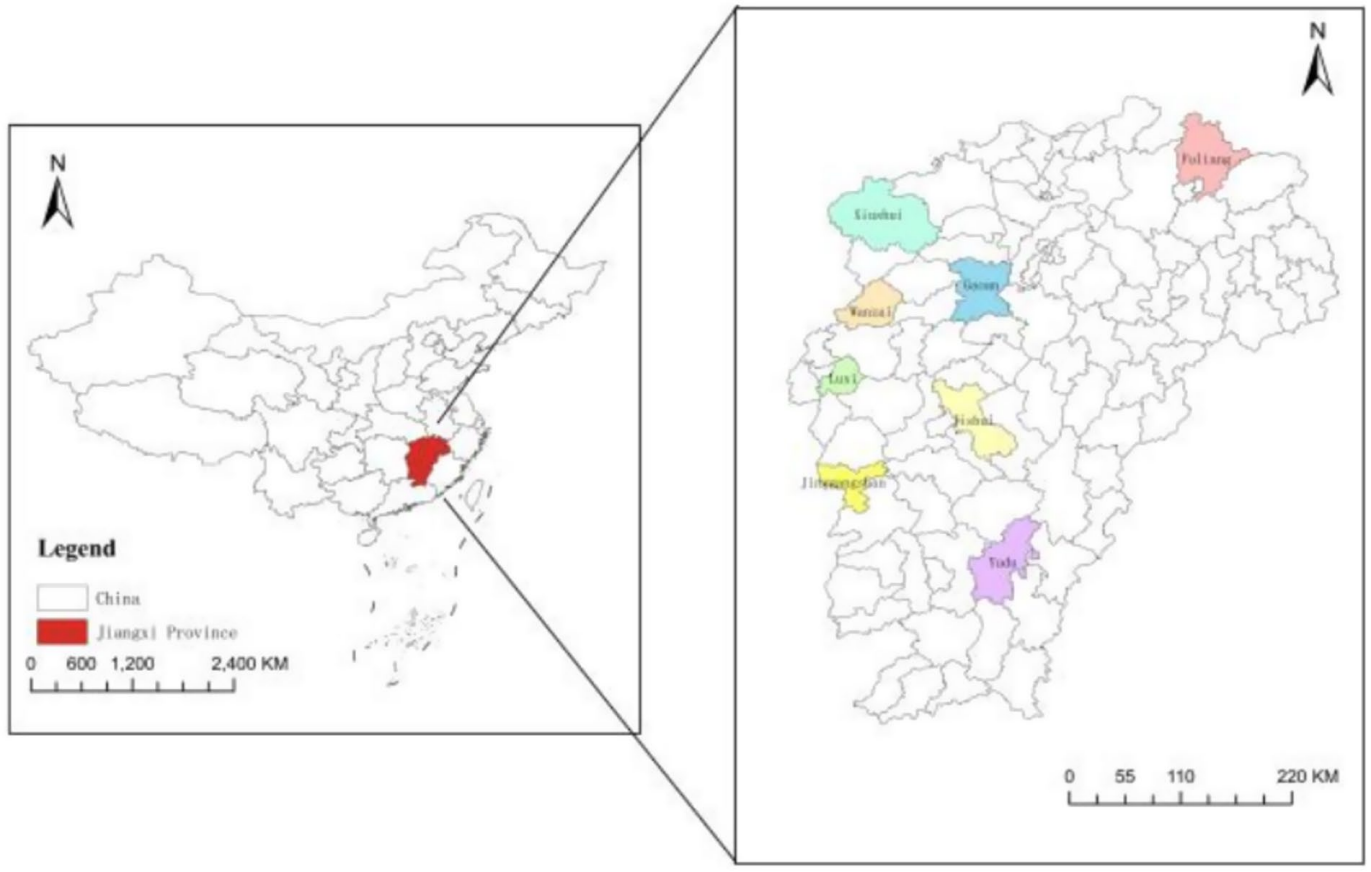
Study area.

This study utilizes field survey data collected by Jiangxi Agricultural University between July and August 2021. To ensure the accuracy and scientificity of the data, the overall sample sampling plan adopts the principle of combining the multi-stage sampling method and the stratified random sampling method, and randomly selects survey locations from Jiangxi Province based on per capita industrial added value. The reason for choosing this indicator is that it can effectively reflect the regional industrialization level and rural economic foundation; the economic foundation directly affects the policy input, infrastructure support, and farmers’ participation capacity in rural human settlement environment improvement, and stratification based on this indicator can cover rural areas at different development stages. Ultimately, 65 natural village groups across eight counties (or cities) in Jiangxi Province were identified (Figure 2). The selected counties encompass northern, central, and southern regions of the province, providing a substantial level of representativeness for the study. The survey employed a random sampling approach to distribute questionnaires to farming households. Each household designated one family member as the respondent. Data were collected through in-person interviews to guarantee precision and comprehensiveness. A total of 700 questionnaires were distributed, with 652 successfully collected, achieving a recovery rate of 93.14%. After excluding questionnaires with incomplete responses, 512 valid samples were retained for analysis, meeting the requirements of this study.

#### 3.2.2 Descriptive statistics

The meaning of each variable and its descriptive statistics are shown in Table 1. The mean value of farmers’ habitat improvement behavior is 4.176, indicating that farmers are enthusiastic about environmental improvement. The mean value of the social networks is 0.683, suggesting that farm households have a more cordial social networks. Among the mediating variables, the mean values of ecological cognition and place attachment are 0.533 and 0.694, respectively, signifying a comparatively high degree of ecological cognition among farm households and an elevated overall level of place attachment. Among the control variables, the sample respondents were primarily male, with a mean age of about 54 years old, mainly with junior high school education, good health, a mean household size of about six people, average economic status, village roads were mainly hardened roads, good hygiene, and the terrain was primarily mountainous and hilly.

**Table 1:** Descriptive statistics for each variable.

Variable type	Variable name	Variable definition and assignment	Average value	Standard deviation
Explained variable	Habitat improvement behaviors of farm households	Participation 0 = 0; Participation 1 = 1; Participation 2 = 2; Participation 3 = 3; Participation 4 = 4; Participation 5 = 5	4.176	1.038
Core explanatory variable	Social networks	Values calculated by the entropy method (see Table 2 for details)	0.683	0.381
Mechanism variables	Ecological cognition	Values calculated by the entropy method (see Table 2 for details)	0.533	0.254
	Place attachment	Values calculated by the entropy method (see Table 2 for details)	0.694	0.151
Control variable	Gender	Female = 0; Male = 1	0.559	0.497
	Age	Age of respondent, years	53.754	14.604
	Educational level	Elementary school and below = 1; Middle school = 2; High school or junior college = 3; College = 4; Bachelor’s degree and above = 5	1.793	0.945
	Health status	Very unhealthy = 1; Less unhealthy = 2; Fair = 3; More healthy = 4; Very healthy = 5	3.729	0.996
	Household size	Number of persons in household, persons	5.613	2.421
	Family economic situation	What is the economic status of your household in the village? Very poor = 1; poorer = 2; average = 3; better = 4; very good = 5	2.902	0.664
	Road condition	Unhardened = 1; Hardened = 2; Tarmac = 3	2.412	0.493
	Hygienic condition	Very poor = 1; poorer = 2; fair = 3; better = 4; very good = 5	4.170	0.623
	Village topography			
	Mountains	(Plain reference) No = 0; Yes = 1	0.307	0.462
	Hills	(Plain reference) No = 0; Yes = 1	0.594	0.492
Area dummy variable (Control group: Ganbei)	Guanzhong	No = 0; Yes = 1	0.125	0.331
	Gannan	No = 0; Yes = 1	0.260	0.439

The descriptions and weights of the core explanatory and mediating variables are detailed in Table 2. Table 3 presents the mean differences of key variables in relation to farm households’ environmental remediation behaviors. For analysis, social networks, ecological perceptions, and place attachments were categorized into low- and high-value groups according to their respective sample means. The results indicate that households in the high-value groups exhibit a greater degree of environmental remediation behaviors compared to those in the low-value groups. This preliminary finding suggests a positive correlation between farm households’ social networks, ecological cognition, place attachments, and their participation in habitat improvement behaviors, providing a foundation for the empirical research in this study.

**Table 2:** Empowerment results of social networks, ecological perceptions and place attachments.

Variable name		Weights	Variable definition and assignment	Average value	Standard deviation	Weights
Social networks	Neighborhood relationships	0.085	What is the status of your neighborhood? Very poor = 1; Poor = 2; Fair = 3; Better = 4; Very good = 5	3.994	0.643	0.085
	Cadre relationship	0.915	Do village officials regularly visit homes to publicize policies and listen to public opinion? No = 0; Yes = 1	0.680	0.467	0.868
			How satisfied are you with the organizational skills and attitudes of village officials? Very dissatisfied = 1; Quite dissatisfied = 2; Generally satisfied = 3; Quite satisfied = 4; Very satisfied = 5	3.645	0.827	0.132
Ecological cognition	Concept cognition	0.750	How well do you understand Habitat for Humanity? Very unaware = 1; Quite unaware = 2; Fair = 3; Quite aware = 4; Very aware = 5	2.680	1.447	0.722
			How much do you know about waste separation? Very unaware = 1; Quite unaware = 2; Fair = 3; Quite aware = 4; Very aware = 5	3.496	1.270	0.278
	Value cognition	0.140	Do you think that participating in remediation helps to promote good health? Strongly disagree = 1; Quite disagree = 2; Fairly = 3; Quite agree = 4; Strongly agree = 5	4.043	0.744	0.605
			Do you think that participating in the remediation will help to protect the ecological environment around our village? Strongly disagree = 1; Quite disagree = 2; Fairly = 3; Quite agree = 4; Strongly agree = 5	4.225	0.676	0.395
	Responsibility cognition	0.110	Are you willing to pay a certain reasonable fee? Very reluctant = 1; Quite reluctant = 2; Fair = 3; Quite willing = 4; Very willing = 5	3.742	0.889	0.613
			Are you willing to persuade your relatives and neighbors to participate in Habitat for Humanity? Very reluctant = 1; Quite reluctant = 2; Fair = 3; Quite willing = 4; Very willing = 5	3.863	0.767	0.387
Place attachment	Village identity	0.320	Would you be willing to tell others about your village? Very reluctant = 1; Quite reluctant = 2; Fairly = 3; Quite willing = 4; Very willing = 5	3.814	0.813	0.320
	Village Pride	0.276	Do you feel proud to be from your village? Very not proud = 1; Quite not proud = 2; Fair = 3; Quite proud = 4; Very proud = 5	3.795	0.766	0.276
	Village satisfaction	0.404	How satisfied are you with the social security situation in your village? Very dissatisfied = 1; Quite dissatisfied = 2; Fair = 3; Quite satisfied = 4; Very satisfied = 5	4.051	0.630	0.404

**Table 3:** Analysis of differences in the means of the variables.

Variable name	Social networks		Ecological cognition		Place attachment		Gender		Economic situation		Village topography	
	Low	High	Low	High	Low	High	Female	Male	Low	High	Non-plain	Plain
Habitat improvement behaviors of farm households	3.915	4.299	3.951	4.415	4.009	4.300	4.053	4.273	4.025	4.221	4.174	4.196

To further explore differentiated characteristics within the data, group analyses were conducted based on the gender of the household head, economic status of the household, and village topography. Economic status was divided into low- and high-value groups using the mean value as the threshold (Table 3). The results indicate that the participation rate of male rural residents in rural human settlements improvement is approximately 5.43 percentage points higher than that of female rural residents. The participation rate of rural residents in the high economic status group in rural human settlements improvement is about 4.87 percentage points higher than that of those in the low economic status group. The participation rate of rural residents in plain areas in rural human settlements improvement is roughly 0.53 percentage points higher than that of rural residents in non-plain areas. Further analysis shows that when social networks are higher than the sample mean, the participation of male rural residents in environmental improvement increases by approximately 10.46 percentage points, and this increase is about 2.34 percentage points higher than that of female residents. For residents in the high family economic status group, their participation in environmental improvement increases by approximately 16.16 percentage points when their social networks exceed the sample mean, and this increase is about 9.31 percentage points higher than that of residents in the low family economic status group. For residents in plain areas, their participation in environmental improvement increases by approximately 12.05 percentage points when their social networks exceed the sample mean, and this increase is about 2.46 percentage points higher than that of residents in non-plain areas. This lays the foundation for the heterogeneity analysis in the subsequent sections.

## 4 Results and discussion

### 4.1 Benchmark regression analysis

To address potential multicollinearity issues among variables, a covariance diagnosis was conducted before the regression analysis. The diagnostic results indicate that the maximum variance inflation factor (VIF) value among the variables is 3.69, while the average VIF is 1.70. All VIF values are well below the threshold of 10, confirming that multicollinearity is not a concern in this study. Using survey data from 512 rural households in Jiangxi Province, this study employs a multivariate ordered probit model to empirically analyze the impact of relational networks on rural habitat improvement behaviors. The regression results are presented in Table 4.

**Table 4:** Regression results of relational networks on rural habitat improvement behavior.

Variable name	Model 1		Model 2		Model 3		Model 4	
	Coefficient	Robust standard error	Coefficient	Robust standard error	Coefficient	Robust standard error	Coefficient	Robust standard error
Social networks	0.365***	0.135	0.345***	0.126	0.364**	0.153	0.371***	0.135
Gender	0.218*	0.113	0.172*	0.101	0.185	0.129	0.219*	0.113
Age	−0.001	0.005	−0.002	0.004	−0.006	0.006	−0.001	0.005
Educational level	0.129*	0.071	0.096*	0.057	0.151*	0.087	0.129*	0.071
Health status	−0.054	0.057	−0.051	0.051	−0.063	0.066	−0.053	0.057
Household size	0.009	0.022	0.010	0.018	−0.008	0.026	0.008	0.022
Family economic situation	0.218**	0.087	0.186**	0.078	0.253**	0.101	0.213**	0.087
Road condition	0.046	0.148	0.041	0.123	0.205	0.168	0.048	0.148
Hygienic condition	0.261***	0.093	0.220***	0.076	0.305***	0.105	0.261***	0.093
Garbage collection facilities							−0.523	0.515
Mountains	0.167	0.206	0.119	0.174	−0.090	0.254	0.166	0.206
Hills	−0.010	0.180	−0.019	0.153	−0.235	0.231	−0.011	0.180
Area dummy variables	Controlled		Controlled		Controlled		Controlled	
Pseudo *R*^2^	0.048				0.058		0.048	
*R*-squared			0.113					
_cons			2.202***	0.555				
Obs	512		512		415		512	

Model 1 is the baseline regression result; Model 2 is the robustness test result of the substitution method model; Model 3 is the robustness test result of changing the sample size; Model 4 is the robustness test result of the supplementary control variables. *, **, *** denote significant at the 10, 5, and 1% levels, respectively; robust standard errors are in parentheses.

In Model 1 of Table 4, the social networks variable is positively associated with rural habitat improvement behaviors and is statistically significant at the 1% level. This finding suggests that stronger social networks among farmers significantly motivate their active participation in improving rural habitats, thus supporting Hypothesis 1. The robustness of this conclusion is further confirmed through alternative methodological models, adjustments to the sample size, and the inclusion of additional control variables. This effect can be attributed to the unique dynamics of acquaintance-based rural societies, where interpersonal relationships and social responsibility play pivotal roles. Farmers with strong neighborly relations often exhibit effective communication and organizational skills. Through interactions with peers, they gain environmental knowledge and are more inclined to participate in remediation efforts under the influence or leadership of others. These individuals also serve as advocates, encouraging others who have not yet participated in environmental protection initiatives to get involved. Similarly, the close ties between village cadres and the farming community further reinforce participation. Cadres play a critical role through demonstrations and policy advocacy, mobilizing farmers to engage in environmental remediation. When farmers feel supported and recognized by village cadres during the remediation process, their trust and alignment with village leadership are strengthened. This mutual support fosters a sense of civic responsibility, prompting farmers to make greater efforts toward the collective environmental management of their village.

In Model 1, gender, educational level, household economic status, and hygienic condition were found to be significant factors influencing farmers’ participation in habitat improvement (Table 3). Male rural residents demonstrated greater participation compared to females. This can be attributed to traditional societal norms, where women often focus more on family-related responsibilities, while men are more inclined to assert their influence in public spheres, such as engaging in community-level activities like environmental remediation. Educational level also showed a favorable and substantial effect on participation. Farmers possessing advanced knowledge tend to exhibit stronger environmental awareness and a heightened sense of responsibility. These individuals are more likely to value the quality of their village environment and take proactive measures to improve it. Household economic status positively influenced habitat improvement behaviors at the 5% significance level. This indicates that wealthier households are more motivated to participate in environmental improvements. Higher economic standing generally correlates with a stronger desire for a high-quality living environment and the financial capacity to support environmental remediation efforts. The sanitary state of the village was significant at the 1% level, exhibiting a positive coefficient. Better hygienic conditions enhance farmers’ comfort and contribute to their physical and mental well-being. This fosters a collective mindset that “environmental protection is a shared obligation,” further motivating participation in habitat improvement activities.

### 4.2 Endogeneity test analysis

Table 5 illustrates the two-stage least squares (2SLS) test results. First, the Hausman test was used to obtain the rejection of the original hypothesis with a probability of *p* = 0.034, which excludes the possibility that the relational network is an exogenous variable, thus necessitating the endogeneity test. The regression results of the first stage show that the coefficient of instrumental variables is positive, which significantly affects the social networks of farmers at the 1% level. The *F* value of the first stage = 42.831, which is greater than the safety valve value of more than 10 according to the empirical criterion, and it can refute the original hypothesis of “the existence of weak instrumental variables,” which indicates that the selection of instrumental variables of the study is more reasonable and consistent with the endogenous explanatory variables. In the second-stage regression results, the social networks is still significantly positive at the 5% level for farmers’ participation in environmental improvement behavior, which confirms the robustness of the baseline regression results (Table 4), which shows that after overcoming the potential endogeneity problem by using the instrumental variable method, the social networks still promotes the participation of farmers in human environment improvement significantly.

**Table 5:** Results of endogeneity test of relational networks on rural habitat improvement behavior.

Variable name	Phase I	Phase 2
	Social networks	Habitat improvement behaviors of farm households
Instrumental variable	0.751*** (0.115)	
Social networks		1.628** (0.646)
Control variable	Controlled	
Hausman test *p*-value	0.034	
*F*-value	42.831	
Obs	512	

**, *** denote significant at the 5, and 1% levels, respectively; robust standard errors are in parentheses. Robust standard errors in parentheses.

Although this study has solved the bidirectional causal endogeneity problem between “social networks” and “farmers’ participation in rural human settlement environment improvement” through the instrumental variable method, it is still necessary to explain the impact of potential omitted variable bias on the model estimation results. First, the benchmark regression has systematically controlled for key variables such as individual characteristics (gender, age, education level, health status), household characteristics (household size, economic status), and village and regional characteristics (road conditions, sanitation conditions, terrain type, regional dummy variables) (Table 1), greatly reducing the risk of systematic omission; Second, in the robustness test, after supplementarily controlling for the potential omitted variable of “completeness of village garbage collection facilities,” the coefficient (0.371) of the core explanatory variable “social networks” shows almost no difference from that of the benchmark regression (0.365) and remains significantly positive (Table 4, Model 4), indicating that detailed variables have extremely low interference with the core conclusions. In conclusion, this study has mitigated potential omitted variable bias to the greatest extent, and the core conclusion that “social networks significantly promote farmers’ participation in rural human settlement environment improvement” is robust.

### 4.3 Heterogeneity analysis

Although social networks can substantially enhance farmers’ involvement in habitat remediation, the influence of social networks on farmers’ involvement in habitat remediation may vary depending on the characteristics of individuals, households and villages. Therefore, this study grouped the samples by gender of farm households, household economic status and village topography to obtain more detailed research conclusions.

#### 4.3.1 Analysis of gender heterogeneity

This study categorizes rural residents into female and male groups, the specific results are shown in Table 6: Compared with the female group, the social networks of the male group exert a more pronounced influence on enhancing their participation in the improvement of the rural habitat environment, the possible reason is that the male rural residents are working outside all the year round, and they have seen the beautiful environment of the city, and they have a stronger desire to improve the environment of the village area, coupled with the limitation of time and space, the male rural residents are lacking in communication and exchanges with their neighbors, In addition, due to time and space constraints, male rural residents lack communication with neighbors and cadres, so when their social networks is improved, the promotion effect of the social networks will be more apparent, and the possibility of their participation in environmental remediation will be significantly increased.

**Table 6:** Results of the analysis of the heterogeneity of the rural population in terms of gender, household economic status and village topography.

Variable name	Female	Male	Low value group	High value group	Non-plain	Plain
Social networks	0.303 (0.199)	0.526*** (0.191)	0.233 (0.310)	0.412*** (0.153)	0.371*** (0.142)	−0.171 (0.535)
Control variable	Controlled		Controlled		Controlled	
Pseudo *R*^2^	0.049	0.061	0.087	0.040	0.054	0.183
Obs	226	286	119	393	461	51

*** denote significant at the 1% levels; robust standard errors are in parentheses.

#### 4.3.2 Heterogeneity analysis of household economic status

In this study, the samples were divided into two groups, the low-value group and the high-value group, based on the mean value of the economic status of the farmers’ households. For farmers with better economic status, the influence of the social networks on their involvement in habitat enhancement is more prominent (Table 6), which may be because farmers with better economic status tend to possess a heightened sense of social responsibility. They may be more concerned about the environment of the village and the public interest. At the same time, participation in habitat improvement requires a certain amount of material and financial resources, and farmers with better economic status usually have more muscular economic strength and willingness to invest (Afroz et al., 2009). Therefore, with the impetus of their social networks, they may be more likely to form common goals and visions, thus be more actively involved in habitat improvement and contribute to the environmental improvement of their villages.

#### 4.3.3 Analysis of topographic heterogeneity of villages

In this study, farmers were categorized into plains and non-plains groups based on the topography of their villages. Compared with the plains farmers, the improvement of the social networks is more likely to foster the participation of non-plains farmers in rural habitat improvement (Table 6), which may be because, compared with the plains, the terrain of the non-plains is complicated. Transportation is inconvenient, which, to a certain extent, increases the difficulty and cost of habitat improvement (Xu and Xia, 2023). In this case, linkages and collaboration among farmers and between farmers and local village committees are critical, and improved relational networks can help strengthen such linkages and optimize the allocation of resources so that remediation efforts can be more effectively promoted.

### 4.4 Analysis of impact mechanisms

Building on the theoretical framework, this study examines whether relational networks promote farmers’ participation in habitat improvement by enhancing their ecological cognition and place attachment. The empirical results are presented in Table 7.

**Table 7:** Results of the mediating effect test for ecological perceptions and place attachment.

Variable name	Model 1	Model 2	Model 3	Model 4	Model 5
	Habitat improvement behaviors of farm households	Ecological cognition	Habitat improvement behaviors of farm households	Village emotions	Habitat improvement behaviors of farm households
Social networks	0.365*** (0.135)	0.083*** (0.026)	0.306** (0.138)	0.097*** (0.018)	0.280** (0.139)
Ecological cognition			0.830*** (0.231)		
Place attachment					0.924*** (0.353)
Control variable	Controlled	Controlled	Controlled	Controlled	Controlled
Bootstrap test		(0.013, 0.108)		(0.016, 0.163)	
Obs	512	512	512	512	512

**, *** denote significant at the 5, and 1% levels, respectively; robust standard errors are in parentheses.

First, the potential mediating role of ecological cognition was tested. In Models 1 and 2, relational networks were found to positively influence both farmers’ environmental remediation behaviors and their ecological cognition, with significance at the 1% level. This indicates that improvements in relational networks not only encourage farmers to engage in environmental remediation but also enhance their ecological awareness. In Model 3, both relational networks and ecological cognition were shown to significantly increase the likelihood of farmers participating in environmental remediation. These findings indicate that ecological cognition serves as a partial mediator in the relationship between relational networks and habitat improvement behaviors. To ensure robustness, a Bootstrap test was conducted, confirming the mediating effect with a confidence interval that excludes zero. The mediating effect of ecological cognition constitutes 18.9% of the total effect, thereby validating Hypothesis 2. Social networks provide a carrier for information transmission to enhance farmers’ ecological cognition. In neighborhood relationships, farmers share practical experiences such as garbage disposal and toilet renovation through daily interactions, transforming abstract environmental protection knowledge into perceivable life cases and gradually clarifying the connection between environmental regulation, their own health, and quality of life; In cadre-mass relationships, village cadres transform environmental policy provisions into plain expressions that align with farmers’ cognitive habits through methods such as door-to-door policy promotion and village affairs disclosure; at the same time, due to the trust endorsement from cadres, farmers’ acceptance and recognition of policies are significantly improved. This social network-based information transmission effectively fills the gaps in farmers’ ecological knowledge and policy understanding, transforming their ecological cognition from “vague perception” to “clear cognition.” After farmers clarify the necessity of environmental regulation and their own responsibilities, they will proactively transform their cognition into participation behavior, thereby forming a complete transmission chain of “social networks → improved ecological cognition → participation in human settlement environment improvement”; this actual functional process confirms the validity of the mediating effect of ecological cognition.

Second, the mediating role of place attachment was examined. Models 1 and 4 reveal that relational networks significantly and positively influence both farmers’ environmental remediation behaviors and their place attachment, with both effects significant at the 1% level. This illustrates that enhanced relational networks not only foster environmental remediation efforts but also strengthen farmers’ emotional ties to their village. In Model 5, both relational networks and place attachment were found to significantly increase farmers’ likelihood of participating in environmental remediation. These results indicate that place attachment partially mediates the relationship between relational networks and habitat improvement behaviors. The Bootstrap test further supports this finding, showing that the mediating effect of place attachment constitutes 24.6% of the total effect. Thus, Hypothesis 3 is confirmed. By establishing high-frequency interactions and trust bonds, social networks provide a practical foundation for the formation of place attachment. In neighborhood interactions, farmers gradually develop a sense of belonging to the village community by jointly participating in village public affairs and sharing production and living resources; In cadre-mass interactions, village cadres’ responses to farmers’ demands and transparent communication on village development plans enable farmers to truly perceive the close connection between themselves and village development, thereby generating a sense of pride and satisfaction toward the village. This trust and emotional connection derived from interactions is not an abstract psychological tendency, but a concrete accumulation of emotions rooted in daily social interactions — because farmers are familiar with their neighbors and trust the cadres, they are more likely to develop an emotional identification of “home” toward the village. After the formation of this place attachment, which encompasses identification, pride, and satisfaction, farmers will spontaneously regard the village environment as an “extension of their own interests,” proactively abandon the tendency to prioritize individual interests, and actively participate in environmental regulation to protect the village’s collective environment. This realizes the transmission of “social networks → place attachment cultivation → participation in human settlement environment improvement,” and this functional logic based on actual emotional accumulation validates the establishment of the mediating effect of place attachment.

### 4.5 Extended analysis

This study further explores the disparities in the impact of neighborhood and community relations on the environmental remediation behavior of farm households. The findings are presented in Table 8. The regression and marginal effect analyses indicate that the cadre-mass relationship is more effective than the neighborhood relationship in prompting farmers to participate in environmental remediation.

**Table 8:** Results of the analysis of the differences in the specific behaviors of neighbor and cadre relations on rural habitat improvement.

Variable name	Model 1	Model 2	Model 3	Model 4
Neighborhood relationships	0.201** (0.079)		0.194** (0.079)	0.071** (0.029)
Cadre-mass relationships		0.310*** (0.116)	0.300** (0.116)	0.110*** (0.042)
Control variable	Controlled			
Pseudo *R*^2^	0.047	0.048	0.052	
Obs	512			

**, *** denote significant at the 5, and 1% levels, respectively; robust standard errors are in parentheses. The explanatory variables in model 4 are the marginal effect results at the “Participation 5” level.

The possible reason is that village cadres are generally local residents, as the dominant force in rural public affairs, closely related to governmental organizations while rooted in the countryside, and their identity is so unique that their behavioral decisions will be paid attention to by members in the village (Chen, 2015). Furthermore, during the environmental remediation process, the propaganda of environmental policies and knowledge is mainly organized by village cadres, so compared with the neighbor relationship, the cadre-mass relationship plays a more prominent role in enhancing the participation of farmers in environmental remediation.

### 4.6 Discussion

This study confirms that social networks have a significant positive promoting effect on farm households’ participation in rural human settlements improvement, and the promoting effect of cadre-mass relations is significantly stronger than that of neighborhood relations. This result is consistent with the conclusions of Teng et al. (2024) and Wang et al. (2022), which further verifies the applicability of the social embedding theory in rural environmental governance scenarios. However, this study further breaks through the limitations of existing literature: most studies regard social networks as a single-dimensional variable. For example, Xu and Xia (2023) only explored the role of clan networks and failed to distinguish the differences between different social networks. In contrast, this study subdivides social networks into neighborhood relations and cadre-mass relations using the entropy weight method, and finds that the impact of cadre-mass relations is more prominent. The core reason for this difference lies in that village cadres, as a bridge between the government and farmers, not only undertake the functions of publicizing environmental policies and organizing improvement activities, but also their behaviors themselves have a demonstration effect. Neighborhood relations mainly spread environmental protection experience through the “peer effect,” but lack policy-level guidance and resource support, resulting in a weaker effect intensity. This finding supplements the segmented research of social capital theory in the field of rural environmental governance, and clarifies the key value of “cadre-mass relations” as a special type of social capital.

In this study, part of the empirical results of control variables echo existing studies, while another part show differences, which provides a new perspective for understanding the influencing factors of farm households’ environmental protection behaviors. From the perspective of consistency, the participation rate of male farmers is higher than that of female farmers, which is consistent with the research conclusions of Zhang and Zhao (2019); Educational level has a positive impact on participation behavior, which is consistent with the findings of Xu and Xia (2023). From the perspective of differences, the family economic status has a significant positive impact on farmers’ participation behavior, which is contrary to the results of Jia et al. (2021), but consistent with the results of Jiao et al. (2024) and Xu et al. (2023). This difference may stem from the economic characteristics of the research areas: in the survey samples from Jiangxi Province, high-income farmers mostly have a certain ability to bear material and time costs, and have higher demands for the quality of living environment, so they are more likely to participate in regulation activities; while the research area of Jia et al. (2021) is rural areas in Northwest China, where farmers’ economic income mostly depends on traditional agriculture and their disposable resources are limited, resulting in the insignificant impact of economic level on participation behavior.

Although Zhang et al. (2023) and Jian et al. (2024) have mentioned the connection between social networks and farmers’ environmental protection behaviors, they have not deeply explored the intermediate pathways of “how social networks are transformed into participation behaviors.” This study systematically verified the partial mediating roles of ecological cognition and place attachment in the impact of social networks on farmers’ participation behavior: the mediating effect of ecological cognition accounts for 18.9% of the total effect, and the mediating effect of place attachment accounts for 24.6%, both of which have passed the Bootstrap test.

The heterogeneity analysis of this study shows that there are significant group differences in the impact of social networks on farmers’ participation behavior: the willingness to participate of male farmers, high-income farmers, and farmers in non-plain areas is more strongly affected by social networks, while the impact on female farmers, low-income farmers, and farmers in plain areas is not significant or weaker. Existing studies rarely conduct subgroup analysis on these groups, and the findings of this study provide an important basis for targeted policy-making.

## 5 Conclusions and recommendations

### 5.1 Conclusion

Improving rural habitats is essential for advancing the comprehensive development of rural areas and enhancing the quality of life for farmers. Among the numerous factors influencing rural habitat improvement behaviors, the role of relational networks, as a fundamental component of social structure, is particularly significant and cannot be overlooked.

This study, based on microdata from 512 rural households in Jiangxi Province, employed an ordered probit model to analyze the impact of relational networks on farmers’ engagement in environmental remediation. It also delved into the intrinsic mechanisms and pathways underlying this relationship.

The findings indicate that relational networks significantly encourage farmers to actively engage in environmental remediation. Among these networks, cadre-mass relationships exhibit a more substantial impact compared to neighborhood relationships. This result remains robust even after undergoing robustness checks and addressing potential endogeneity concerns. Furthermore, the effect of relational networks on participation is more pronounced among specific groups, such as men, rural residents with higher economic status, and those living in non-plains areas.

In addition, the study highlights that ecological cognition and place attachment serve as partial mediators in the relationship between relational networks and farmers’ participation in environmental remediation. These mediating factors underscore the pathways through which relational networks influence farmers’ environmental behaviors, illuminating the complex interplay between social structure and individual action.

These findings emphasize the importance of strengthening relational networks, fostering ecological awareness, and cultivating a strong sense of community to promote rural habitat improvement initiatives effectively. By addressing these dimensions, policymakers can create more targeted and sustainable strategies for rural development.

### 5.2 Recommendations

To play the auxiliary role of neighborhood relations, the “Neighborhood Joint Cleaning Day” activity is carried out at the natural village level: one fixed day per month is set to organize farmers to participate in public area cleaning on a household basis, and sessions of “farm tool sharing” and “skill exchange” are arranged after the activity; For natural villages with a participation rate exceeding 80%, county-level agricultural and rural departments provide material rewards (one sorted trash bin and one set of disinfection supplies per household). Meanwhile, participation is incorporated into the evaluation indicators for the “Civilized Family” selection to strengthen neighborhood collaboration awareness. To exert the key promoting role of cadre-mass relations, the “village cadre area responsibility + monthly home visit” system is implemented at the administrative village level: It is clarified that members of the village Party and government committees divide responsibility areas according to residential areas, and conduct home visits to farmers in their responsible areas at least twice a month. They focus on recording environmental improvement demands (such as garbage collection frequency, sewage pipe network maintenance, etc.), and synchronize the “demand list – handling progress – completion results” on the village public notice board and WeChat groups to ensure a closed-loop response within 15 working days; An “Environmental Improvement Consultation Meeting” is held every quarter, where farmhouse representatives are invited to participate in program voting (such as the schedule of collective cleaning activities, selection of waste classification facilities). For reasonable suggestions put forward by farmers (such as adding garbage collection points in mountainous areas), township governments provide special funds to support their implementation, so as to improve cadre-mass trust.

A cultivation system featuring “popularized publicity + practical training + participatory enhancement” is established: the county-level Bureau of Agriculture and Rural Affairs, in collaboration with the Environmental Protection Bureau, sets up an “Ecological Cognition Promotion Team” and designs hierarchical content targeting farmers’ cognitive weaknesses. For conceptual cognition (e.g., policies on human settlements improvement), a combined model of “mobile publicity vehicles + village radio” is adopted, and graphic guide cards are simultaneously posted on village public notice boards; For value cognition, “farmers’ experience-sharing sessions” are held in each village, where farmers who have participated in the improvement are invited to share the benefits they have gained from participating in environmental improvement; For responsibility cognition (e.g., active participation, mobilizing others), a “cognition-practice linkage mechanism” is implemented: farmers can earn “environmental protection points” by attending one garbage sorting training session or collective cleaning activity. These points are publicly announced by village groups every month, and special funds from township finances ensure the supply of materials. Meanwhile, a cultivation scenario of “cultural connection + facility support + achievement sharing” is created: taking administrative villages as units, village Party and government committees take the lead in building “village history and culture corners” at village entrances or public activity centers, and organize “elderly villagers telling village history” activities once a quarter to help farmers strengthen their identification with the village through memories; For fostering village pride, a short video on “village improvement before and after comparison” is filmed every 6 months, which is promoted through village WeChat groups and township official WeChat accounts. Meanwhile, an “improvement achievement display wall” is set up in the village, with the names of participating farmers marked to enhance their sense of accomplishment; Focusing on improving village satisfaction, the construction of “miniature livelihood facilities” is prioritized in villages undergoing environmental improvement: one “miniature garbage collection point” is built for every 30 households, and one “miniature leisure square” is built per village. This deepens the emotional bond with the village, thereby encouraging farmers to take the initiative in environmental maintenance.

Activate the demonstration role of male farmers. Select 2–3 male leaders per village (with priority given to capable villagers and veterans) and implement an “environmental protection points system”: 10 points are awarded for participating in one collective improvement activity, and 15 points for mobilizing others; points can be exchanged for chemical fertilizers and seeds; Leaders take the lead in forming volunteer teams to be responsible for hard-to-clean areas such as mountain slopes and steep hills, conducting centralized operations twice a month. “Outstanding Leaders” are selected quarterly, with a reward of 200 yuan and a certificate of honor. Address the difficulty of low-income farmers in participating. Establish a “dual subsidy” mechanism: issue work delay subsidies for participating in improvement activities, and provide sorted trash bins and toilet renovation tools free of charge; Give priority to hiring low-income farmers as village cleaners, whose responsibilities include environmental maintenance, to achieve “income increase through participation”; Conduct skill training once every 6 months, with free lunches provided during the training period. Adapt to the needs of non-plain areas. Divide 3–5 cooperation zones based on townships, assign one full-time coordinator to each zone, and organize cross-village joint dredging and shared pollution control equipment; Give priority to allocating small garbage transfer vehicles (suitable for mountain roads) and build “mountain garbage collection points.”

### 5.3 Limitations of the study

Although this study verified that the social networks can promote the active participation of farmers in rural habitat improvement, it still has the following limitations. On the one hand, this study used cross-sectional data for analysis, which has limitations in dynamic effect analysis and other aspects. Future research could select longitudinal farmer tracking data for analysis. On the other hand, this study only focuses on the green lifestyles of farmers, and future research can study the green production methods of farmers.

## Data Availability

The original contributions presented in the study are included in the article/supplementary material, further inquiries can be directed to the corresponding authors.
